# Assessment of cerebral autoregulation using continuous-wave near-infrared spectroscopy during squat-stand maneuvers in subjects with symptoms of orthostatic intolerance

**DOI:** 10.1038/s41598-018-31685-y

**Published:** 2018-09-05

**Authors:** Jae-Myoung Kim, Jong-Kwan Choi, Mingyu Choi, Minsu Ji, Gunpil Hwang, Sang-Bae Ko, Hyeon-Min Bae

**Affiliations:** 1 School of Electrical Engineering, Korea Advanced Institute of Science and Technology, Daejeon, 34141 Republic of Korea; 2 Department of Neurology, Seoul National University Hospital, Seoul, 03122 Republic of Korea

## Abstract

Orthostatic lightheadedness in healthy young adults often leads to syncope in severe cases. One suggested underlying mechanism of orthostatic lightheadedness is a drop in transient blood pressure (BP); however, a decrease in BP does not always lead to a drop in cerebral blood flow (CBF) due to cerebral autoregulation (CA). We present a direct assessment method of CA using a multichannel continuous-wave near-infrared spectroscopy (CW-NIRS) device that measures the temporal changes in oxy- and deoxy-hemoglobin concentrations in the prefrontal cortex. Twenty healthy young adults were recruited. During the experiment, continuous beat-to-beat BP and heart rate were simultaneously measured during repetitive squat-stand maneuvers. We introduce a new metric termed ‘time-derivative hemodynamic model (DHbT)’, which is the time-derivative of total-hemoglobin concentration change that reflects the changes of cerebral blood volume and CBF. Although the absolute levels and the variations of systolic and diastolic BPs and mean arterial pressure showed no significant difference between the two groups, the proposed model showed a distinct difference in slope variation and response time of DHbT between the subjects with frequent symptom of orthostatic intolerance and the healthy control subjects. Thus, these results clearly demonstrate the feasibility of using CW-NIRS devices as a CA performance assessment tool.

## Introduction

Orthostatic lightheadedness, which occurs upon abrupt standing from supine or squatting positions, is common even in healthy young adults^1^. It may appear as a mild transient orthostatic dizziness, but it can lead to syncope in severe cases. Physiologic monitoring studies using continuous assessment of beat-to-beat blood pressure (BP) and heart rate (HR) have suggested that the underlying mechanism behind orthostatic lightheadedness is a transient BP drop^2–4^. However, a decrease in BP does not always lead to a drop in cerebral blood flow (CBF) when cerebral autoregulation (CA) is intact^5^; CA is a mechanism that maintains a constant CBF regardless of changes in BP^6^. Therefore, a direct monitoring of CBF can be an effective means for evaluating orthostatic intolerance.

Recently, several reports have shown that near-infrared spectroscopy (NIRS) can be used as a tool for non-invasive continuous monitoring of cerebral circulation^7–9^. Changes in oxy- and deoxy-hemoglobin concentrations (HbO_2_ [oxygenated hemoglobin] and HbR [reduced hemoglobin], respectively), measured using NIRS, can be used as surrogates for cerebral hemodynamics.

In this study, we explored the possibility of using NIRS for the assessment of the arterial-cardiac baroreflex function during repeated squat-stand maneuvers. We hypothesized that the posture change would incur hemodynamic changes that reflect a baroreflex mechanism. The NIRS measurements were compared with conventional prognostic indicators such as systematic BP and HR measured using beat-to-beat photoplethysmography.

## Materials and Methods

### Participants

Twenty right-handed healthy young students (male/female, 12/8; age, 22.1 ± 3.4 years) were enrolled from the Korea Advanced Institute of Science and Technology (KAIST). Participants did not have any history of neurological or cardiovascular diseases, all were non-smokers, and none were taking any medications at the time. Prior to the assessment, participants completed a validated questionnaire with a Cronbach’s α score of 0.888 to specify the presence and frequency of the orthostatic intolerance (OI) symptoms^10^. Subjects were categorized into a control group or a symptom group based on the questionnaire symptom score. Subjects with a symptom score below three which were presumed to be healthy individuals unlikely suffer from any kind of orthostatic dysregulation^10^ (control group, n = 6), and those with a symptom score greater than or equal to three were categorized into the symptom group (n = 14). The study was approved by the KAIST institutional review board and performed in agreement with the Declaration of Helsinki. (IRB approval No. KH2015-53). All participants received a complete explanation of the study and signed written informed consent for the study and could withdraw from the study at any time.

### Experimental Design

**Figure 1:**
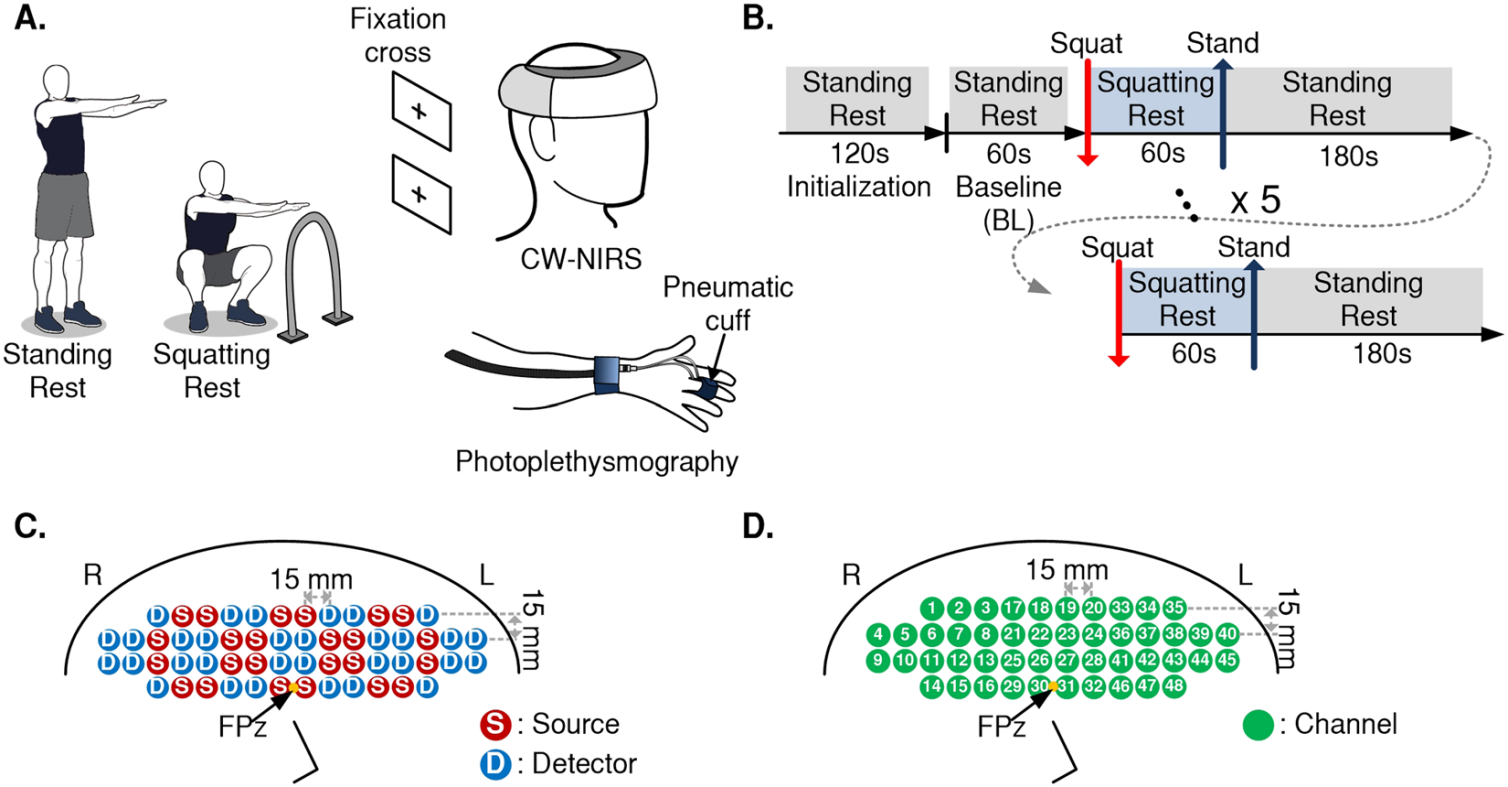
Experimental design. (**A**) Data acquisition setup (**B**) Experimental protocol (**C**) Optode position (**D**) Channel position. R = right; L = left; Fpz = frontal pole zero; CW-NIRS = continuous-wave near-infrared spectroscopy.

Figure 1A shows the data acquisition setup. The changes in optical intensity were measured using a commercial wireless continuous-wave near-infrared spectroscopy (CW-NIRS) system (NIRSIT, OBELAB Inc., Republic of Korea) at a sampling rate of 8.13 Hz. The optical probes consist of 24 sources and 32 detectors. The sources of the CW-NIRS system were dual-wavelength vertical cavity surface emitting laser diodes operating at 780 nm and 850 nm and the detectors were implemented with silicon photodiodes. The output power of the laser diodes was automatically controlled to satisfy the FDA regulations of maximum permissible exposure standards (i.e. under 1 mW) to human subjects. The gain of the detectors was individually calibrated for each subject to maximize the signal intensity without saturation, taking different skin pigmentations, skin and skull thickness, and other biological factors into account. The optical probes were arranged at distances of 1.5 cm as shown in Fig. 1C, where a total of 48 pre-defined channels with a source-detector separation of 3 cm were measured. Such separation is adequate for the depth penetration of 20 mm from the scalp, which is required for the inclusion of the measurements from the microvasculature of the cerebral cortex^11^. The measurements were obtained from the prefrontal cortex (see Fig. 1D), where the center of the lowermost optical probes was aligned to the frontal pole zero (FPz) location of the 10–20 EEG system to remove positional uncertainty between subjects. The device features a rubber cap at the tip of the optical probe to ensure ambient-light-proof contact to the forehead; the device was tightly fastened to minimize additional motion-induced contamination from contact variations.

A continuous beat-to-beat finger photoplethysmography system (Finometer®, Finapres Medical Systems, Amsterdam, Netherlands) was used to measure systolic BP (SBP), diastolic BP (DBP), mean arterial pressure (MAP), and HR non-invasively at a sampling frequency of 1.4 Hz. A pneumatic finger cuff was wrapped around the left middle finger for the measurements. The outputs of the CW-NIRS and the photoplethysmography devices were recorded simultaneously in a dark environment to maintain signal integrity by preventing ambient light during the experiment.

All subjects underwent an identical test protocol composed of series of repetitive maneuvers to trigger CA. Prior to the CW-NIRS and the photoplethysmography measurements, subjects were trained to stand upright and to squat in a uniform manner. The protocol consisted of a two minutes initialization for the CW-NIRS system and the pneumatic cuff for stabilization while the participants were in a standing-rest position, as shown in Fig. 1B. Then, the subjects rested again in the standing-rest position for one minute to record the baseline (BL) levels. Subsequently, they were instructed to gently sit into a squatting position and hold for one minute, during which they could hold onto a preinstalled grab-bar with their right hand to aid balance, so as to minimize motion artifacts caused by abrupt movements. During the squatting-rest posture, subjects could either stand on tiptoe or with their feet flat, depending on their preference. Lastly, the subjects were instructed to return to the standing-rest position and hold for 3 minutes. The entire maneuver (one minute squat followed by three minutes of standing) was repeated five times per subject (see Fig. 1B). The subjects were instructed to breathe normally during the test protocol. During the rest periods, the subjects focused on a black fixation cross that was positioned according to their head position (i.e. during standing or squatting), to minimize hemodynamic changes caused by both cognitive activities and gravitational blood shifting under head tilting^12^. The event markers were simultaneously recorded manually by the observer when the subjects change postures, by using embedded event marking functions in each system.

### Data Processing

Data analysis was performed using MATLAB (R2013b; MathWorks, Natick, USA). Prior to the analysis, the CW-NIRS channels with insufficient signal integrity, i.e., signal-to-noise ratio (SNR) less than 30 dB, were excluded for further analysis. We obtained the mean (S_m_) and standard deviation (S_std_) of the motion-less BL signal (i.e. 10 to 15 seconds) to calculate the SNR (=20·log_10_(S_m_/S_std_)). The signals were then converted into logarithmic changes in optical density. The optical density variations were then filtered using a low-pass filter with a cutoff frequency of 0.2 Hz to suppress the instrumental noise and physiological oscillations (i.e. Mayer’s waves, respiration noise, and heartbeat). These filtered optical density variations were converted to changes in HbO_2_ and HbR using the modified Beer-Lambert law^13^. In this study, the variation in the total hemoglobin concentration (HbT = HbO_2_ + HbR) was the main focus during the squat-stand maneuver because HbT is regarded as a surrogate for cerebral blood volume (CBV)^14–16^.

The raw SBP, DBP, MAP, and HR signals acquired from the photoplethysmography device were filtered using a 4-tap moving average filter to suppress the non-physiological signal caused by instant contact variation during the maneuvers.

For reliability, all the measurements (i.e. HbT, SBP, DBP, MAP, and HR) from the CW-NIRS and the photoplethysmography devices were block-averaged across five maneuver trials per subject.

### Time-Derivative Hemodynamic Model

In this study, a new metric for the assessment of individual CA performance was introduced. The metric, referred to as “DHbT”, the time-derivative of HbT where DHbT = dHbT/dt, was used to quantify HbT variations during the squat-stand maneuvers. For the numerical differentiation, a two-point difference algorithm was used^17^.

**Figure 2:**
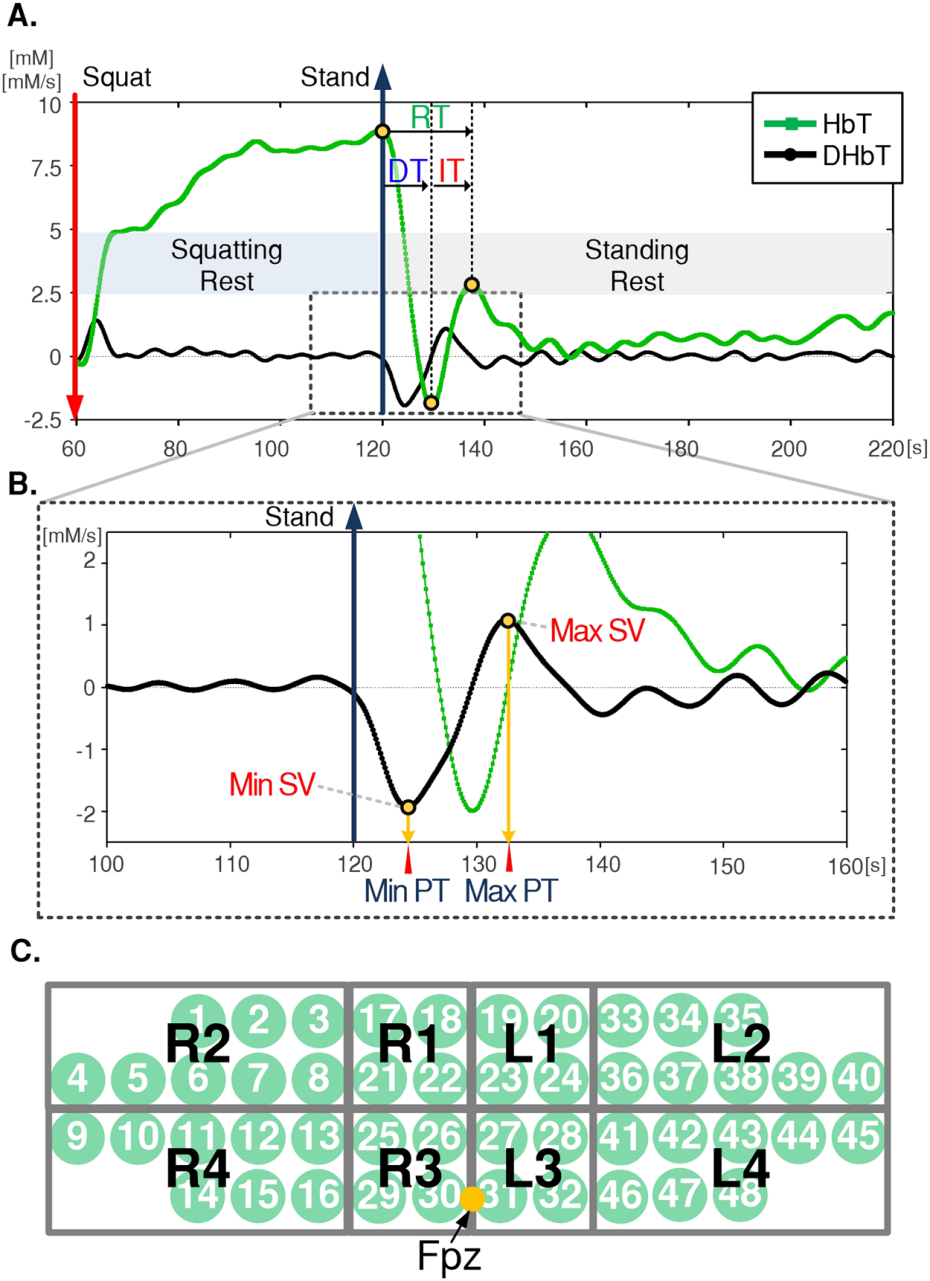
Total hemoglobin (HbT) and time-derivative of HbT (DHbT) change during squat-stand maneuver. (**A**) An example of the block averaged (5-block) HbT change and DHbT with parameters evaluated from HbT; the decrease time (DT), increase time (IT) and recovery time (RT). (**B**) Closer view of DHbT change during the task with objective parameters obtained from DHbT; the maximum and minimum slope variations (Max SV, Min SV) and the peak-time of the slopes (Max PT, Min PT). (**C**) Measured sections from the prefrontal region of the brain (The channels were grouped into eight sections).

A typical change in HbT and DHbT under the squat-stand transition is shown in Fig. 2A. Since HbT variation is tightly coupled with that of CBV^16–18^, we assumed that the hemodynamic recovery response extracted from the HbT is caused by the CA in conjunction with the baroreflex mechanism of any given subject. Since CW-NIRS measures only relative hemodynamic changes from BL, assessment of individual CA performance using only measured HbT is difficult. In order to overcome this limitation, DHbT which translates relative change into absolute numbers, was proposed for assessment of individual CA performance. For this, the absolute value of the maximum and minimum slope variation (Max SV and Min SV, respectively) of DHbT and the corresponding peak-times (Max PT and Min PT, respectively) were extracted as objective parameters as shown in Fig. 2B.

Additionally, in order to compare the feasibility of DHbT with HbT and to provide physiological insight, the decrease time (DT), defined as the time to nadir right after standing, and the increase time (IT), defined as the time to the overshoot peak from the nadir, and the recovery time (RT), defined as sum of DT and IT, were extracted from the transient response of HbT by using the similar approach given in the previous study^19^ as shown in Fig. 2A.

The 48 measured channels of the CW-NIRS device were divided into 8 sections to represent regional HbT and DHbT (as shown in Fig. 2C). Prior to the quantitative analysis, we validated that the transient response of the HbT and DHbT of the entire channels exhibited consistent patterns within each subject, offering a means to measure individual CA characteristics, as in a previous investigation^14^. The signals were grand-averaged for each section, excluding those from rejected channels in order to ensure signal integrity. The channels in each section were assigned as follows: R1 channels: 17, 18, 21, and 22; R2 channels: 1, 2, 3, 4, 5, 6, 7, and 8; R3 channels: 25, 26, 29, and 30; R4 channels: 9, 10, 11, 12, 13, 14, 15, and 16; L1 channels: 19, 20, 23, and 24; L2 channels: 33, 34, 35, 36, 37, 38, 39, and 40; L3 channels: 27, 28, 31, and 32; L4 channels: 41, 42, 43, 44, 45, 46, 47, and 48. Considering the individual differences in forehead size, more channels were assigned for sections R2, R4, L2, and L4; the majority of borderline channels located in the hairy area, left and right temples of the forehead, tended to have low SNR due to poor scalp-optical probe coupling. The HbT and DHbT responses were regionally grand-averaged, and Max SV, Min SV, Max PT, Min PT, DT, IT, and RT values were then obtained for each subject.

### Statistical analysis

We performed statistical analysis using SPSS (IBM Corp. Released 2012, IBM SPSS Statistics for Windows, Version 21.0. Armonk, NY, USA) and GraphPad Prism (GraphPad Prism version 5 for Windows, GraphPad Software, San Diego, CA, USA, www.graphpad.com). Means and standard deviations were extracted from continuous variables, and the Shapiro-Wilk test was used to test normality of the data. Between-groups differences in continuous variables were evaluated using unpaired t-tests (if data were normally distributed) and Mann–Whitney U tests (if data were not normally distributed). Moreover, within-group changes in cardiovascular variables were evaluated using Wilcoxon signed-rank tests. Significance levels were set to P < 0.05.

## Results

### Subject characteristics

Table 1 shows group characteristics, represented by group-averaged BL values and standard deviations. There were no significant between-group differences other than the symptom score (Mann-Whitney U test, P = 0.00001). Although the symptom group tended to have a higher HR at BL, this difference did not reach significance.

**Table 1:** Subject characteristics.

	Control Group (n = 6)	Symptom Group (n = 14)	P between groups
Sex (m/f)	5/1	7/7	
Age	21.5 ± 2.8	22.4 ± 3.8	P = 0.941
BMI (kg/m^2^)	22.6 ± 2.3	22.1 ± 2.2	P = 0.631
SBP (mmHg)	108.2 ± 18.0	104.9 ± 10.1	P = 0.607
DBP (mmHg)	65.5 ± 11.6	64.8 ± 5.0	P = 0.899
MAP (mmHg)	81.9 ± 12.2	80.3 ± 6.0	P = 0.702
HR (bpm)	87.7 ± 8.0	91.8 ± 11.9	P = 0.445
Symptom Score	1.17 ± 0.98	9.79 ± 5.79	**P = 0.00001**

Values are presented as the mean ± SD. Text in bold indicates statistical significance. SD = standard deviation; BMI = body mass index; SBP = systolic blood pressure; DBP = diastolic blood pressure; MAP = mean artery pressure; HR = heart rate.

### Blood Pressure and Heart Rate Variation

**Figure 3:**
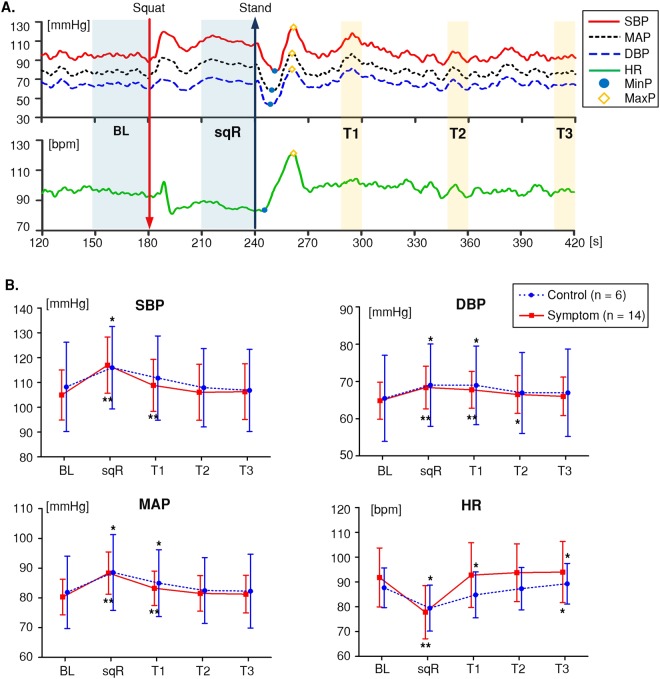
Systolic, diastolic, and mean arterial blood pressure (SBP, DBP, and MAP respectively) and heart rate (HR) during the squat-stand maneuver. Mean and standard deviation values were extracted in each group from the pre-defined time-intervals such as baseline (BL), squatting-rest (sqR), T1, T2, and T3 (150 to 180 seconds, 210 to 240 seconds, 295 to 300 seconds, 355 to 360 seconds, and 415 to 420 seconds, respectively). Initial minimum and maximum peak (MinP and MaxP) of the response immediately after standing. (**A**) An example SBP, DBP, MAP and HR responses measured from the finger artery. (**B**) Between-group comparison of cardiovascular variables. Asterisks: significant differences (*P < 0.05, **P < 0.01) between BL and other time intervals (i.e. sqR, T1, T2, and T3) in each group. Error bars indicate the standard deviation of the mean.

A typical response of the SBP, DBP, MAP, and HR during the maneuver is shown in Fig. 3A. For each subject, BL of SBP, DBP, MAP, and HR values were obtained by averaging the 30-second measurements prior to the squat. Additionally, the block-averaged response was divided into four distinct time intervals to derive a single representative value; one averaged 30-second epoch from the squatting-rest position (sqR) and three averaged 5-second epochs from the standing-rest position (T1, T2, and T3) representing for each of the recovery phases after standing at 60, 120, and 180 seconds. These time intervals were selected to extract the representative values of SBP, DBP, MAP, and HR while the CA progresses into the autoregulatory plateau after the squat or stand. Additionally, from the cardiovascular oscillation right after standing, minimum and maximum peak (MinP and MaxP) of SBP, DBP, MAP, and HR values were obtained for analysis.

The overall pattern of cardiovascular responses during the squat-stand maneuver were similar between the control and symptom groups (Fig. 3B). The control group demonstrated higher BP (SBP, DBP, and MAP) and lower HR in general at each interval than the symptom group. However, these differences did not reach significance at BL, sqR, T1, T2, and T3 (n.s., Bonferroni-corrected Mann-Whitney U tests, significance level adjusted to 0.01 [obtained by 0.05/5]).

The magnitude of SBP and MAP increased significantly at sqR compared to BL for both the control group (7.15% and 8.17% respectively, P < 0.05) and symptom group (11.45% and 9.99% respectively, P < 0.01). For both groups, when the subjects returned to the standing-rest position, SBP and MAP dropped and recovered to the BL level during T2 and T3.

Similar to SBP and MAP, DBP increased significantly after the squat (during the sqR interval) compared to BL, for both the control group (5.41%, P = 0.046) and symptom group (5.49%, P = 0.002). However, the absolute increment of DBP was lower than that of SBP and MAP, and persisted throughout T1 and then recovered to the BL levels for both groups.

Meanwhile, HR fell substantially during sqR compared to BL, for both the control group (−9.36%, P = 0.028) and the symptom group (−15.23%, P = 0.001) and then increased rapidly subsequent to the squat-stand transition (T1, T2, and T3). BP and HR returned to the BL level within T2 following the squat-stand transition and then showed slight increase in T3 compared to BL for both the control group (1.85%, P = 0.046) and the symptom group (2.35%, P = 0.035). However, the block-averaged variations of SBP, DBP, MAP, and HR in response to the orthostatic challenge were not significantly different between groups.

**Figure 4:**
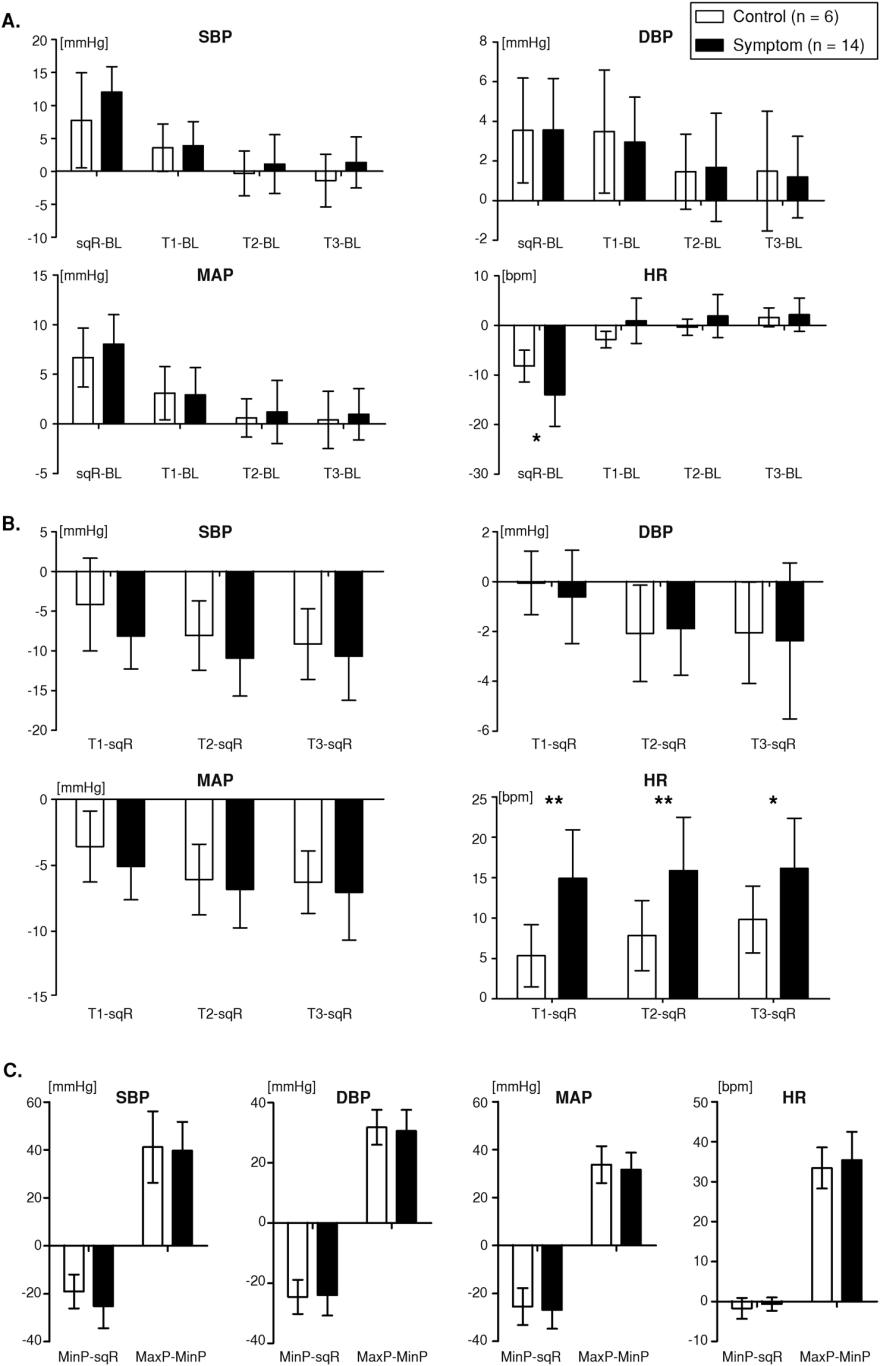
Between-group comparison of cardiovascular responses. Graphs of (**A**) deviation from BL values at sqR, T1, T2, and T3. (**B**) Deviation from sqR values at T1, T2, and T3. (**C**) Transient variation of cardiovascular responses. Asterisks: significant differences (*P < 0.05, **P < 0.01) between groups. Error bars indicate the standard deviation of the mean. BL = baseline; sqR = squatting-rest.

The differences in cardiovascular measures over time (vs. BL) are shown in Fig. 4A. SBP, DBP, and MAP increased from the BL level for both groups as noted above. However, no between-group differences were observed. SBP, DBP, and MAP gradually returned to BL levels over T1, T2, and T3. BP recovery showed no significant between-group difference.

The symptom group demonstrated significant decrease in HR at sqR (vs. BL) compared to the control group (Mann-Whitney U test; control, −8.2 ± 3.2 bpm; symptom, −14.0 ± 6.4 bpm; P = 0.041). Unlike SBP, DBP, and MAP, HR returned immediately to the BL level at T1 and remained unchanged at T2 and T3. There were no between-group differences in HR recovery to the BL level.

The changes in cardiovascular measures at T1, T2, and T3 vs. sqR are shown in Fig. 4B. The drops in SBP, DBP, and MAP seen at T1, T2, and T3 were not significant. Such correspondingly indicates a negative test result of the classical orthostatic hypotension (OH) test, which is defined as an SBP drop of more than 20 mmHg and/or a DBP drop of more than 10 mmHg after three minutes of standing^3,20^. By contrast, the HR level change (vs. sqR) was significantly greater in the symptom group than the control group at T1 (P = 0.003), T2 (P = 0.005), and T3 (P = 0.041). Particularly at T1, the variations in HR (vs. sqR) were 9.6 bpm higher in the symptom group (15.0 ± 6.0 bpm) than the control group (5.3 ± 3.9 bpm). The overall HR deviation from sqR for the control group demonstrated gradual increase over time, whereas the deviation for the symptom group showed no significant variations.

The transient variations in SBP, DBP, MAP, and HR are shown in Fig. 4C. The difference between MinP and sqR (MinP – sqR), which implies the initial transient reduction of the cardiovascular response, was calculated. Additionally, the maximum relative change to the MinP was extracted by taking the difference between MaxP and MinP (MaxP – MinP), as shown in Fig. 3A.

MinP – sqR of SBP, DBP, and MAP were not significantly different between the control and symptom groups. Conversely, the initial transient reduction of BP demonstrated a positive result for a few participants with the conventional initial orthostatic hypotension (IOH) diagnosis (Control 83.3% and Symptom 57.1%); a transient decrease in SBP by more than 40 mmHg and/or DBP by more than 20 mmHg within the first 15 second of standing prior to BP recovery under supine-stand test^4^. In addition, MaxP – MinP of the SBP, DBP, and MAP did not show significant differences between the two groups.

HR remained stable when the subjects returned to the standing position with a slight deviation in MinP – sqR for both groups (control, −1.7 ± 2.6 bpm; symptom, −0.6 ± 1.7 bpm). MaxP – MinP of HR increased abruptly over 30 bpm for both groups (control, 33.4 ± 5.16 bpm; symptom, 35.4 ± 7.1 bpm). However, there was no significant difference in MaxP – MinP of HR between the two groups.

### Cerebral Microvascular Hemodynamic Response

The objective parameters obtained from the regionally grand-averaged DHbT are summarized in Table 2. The squat-stand maneuver resulted in significantly smaller increase in Min SV in section R4 in the symptom group compared to the control group (P = 0.033). However, the variation of the Max SV did not show significant differences in the symptom group compared to the control group.

**Table 2:** DHbT parameters between groups per section.

Section	Parameter		Control	Symptom	P	Section	Parameter		Control	Symptom	P
R1	SV	Max	9.45 ± 4.91	8.62 ± 3.1	1.000	L1	SV	Max	8.8 ± 4.54	8.76 ± 3.02	0.968
		Min	−13.8 ± 7.74	−9.53 ± 2.88	0.274			Min	−12.08 ± 6.48	−10.11 ± 3.21	0.779
	PT	Max	11.41 ± 1.6	13.13 ± 1.39	**0.033**		PT	Max	11.78 ± 1.79	13.13 ± 1.23	0.179
		Min	2.48 ± 0.53	2.94 ± 0.66	0.130			Min	2.29 ± 0.68	2.93 ± 0.71	0.091
R2	SV	Max	8.42 ± 3.77	8.42 ± 3.77	0.353	L2	SV	Max	7.24 ± 3.19	8.4 ± 3.09	0.547
		Min	−13.56 ± 7.18	−9 ± 3.21	0.274			Min	−12.08 ± 7.62	−9.4 ± 3.39	0.602
	PT	Max	11.35 ± 1.48	13.31 ± 1.52	**0.020**		PT	Max	11.33 ± 1.99	13.31 ± 1.3	**0.041**
		Min	2.27 ± 0.29	3.09 ± 0.62	**0.002**			Min	2.54 ± 0.56	2.98 ± 0.49	**0.033**
R3	SV	Max	7.28 ± 2.94	6.1 ± 3.41	0.207	L3	SV	Max	7.57 ± 3.65	7.55 ± 3.42	0.968
		Min	−14.23 ± 7.12	−8.27 ± 3.27	0.051			Min	−13.08 ± 7.13	−10.49 ± 3.63	0.904
	PT	Max	11.78 ± 1.42	13.83 ± 1.24	**0.006**		PT	Max	11.98 ± 1.59	13.55 ± 1.41	**0.033**
		Min	2.23 ± 0.54	3.56 ± 1.02	**0.001**			Min	2.19 ± 0.79	3.63 ± 1.31	**0.009**
R4	SV	Max	7.09 ± 2.7	4.71 ± 3.03	0.153	L4	SV	Max	6.4 ± 2.86	6.73 ± 3.94	0.841
		Min	−14.03 ± 5.44	−8.62 ± 4.1	**0.033**			Min	−13.8 ± 7.97	−10.26 ± 5.62	0.602
	PT	Max	11.82 ± 1.64	13.14 ± 2.19	0.153		PT	Max	11.82 ± 2.04	12.86 ± 2.38	0.312
		Min	2.25 ± 0.17	3.04 ± 0.75	**0.003**			Min	2.23 ± 0.16	3.08 ± 1.56	**0.033**

Values are presented as the mean ± SD unless otherwise specified. P values were calculated using Mann-Whitney U tests. Text in bold indicates statistical significance. The unit of the Max/Min SV are [10–4 mM/s], Max/Min PT are [second]. R = right; L = left; SD = standard deviation; SV = slope variation; PT = peak time.

Moreover, such slope variation appeared with an increased time delay (i.e. Max PT and Min PT) among subjects in the symptom group. The Max PT in sections R1 (P = 0.033), R2 (P = 0.020), R3 (P = 0.006), L2 (P = 0.041), and L3 (P = 0.033) showed significant increase, and the Min PT in the following sections demonstrated significant increases: R2 (P = 0.002), R3 (P = 0.001), R4 (P = 0.003), L2 (P = 0.041), L3 (P = 0.009), and L4 (P = 0.033).

Table 3 summarizes DT, IT and RT values extracted from the grand-averaged HbT change for each section. After squat-stand maneuver, significant increase in DT in symptom group was observed in sections R3 (P = 0.041), R4 (P = 0.02), and L1 (P = 0.033) compared to the control group. Moreover, RT increased significantly in sections R3 (P = 0.033), R4 (P = 0.026), L1 (P = 0.026), L3 (P = 0.015), and L4 (P = 0.009) in the symptom group compared to the control group. However, IT showed no significant differences in all the sections.

**Table 3:** DT, IT, and RT parameters between groups per section.

Section	Parameter	Control	Symptom	P	Section	Parameter	Control	Symptom	P
R1	DT	7.68 ± 1.39	8.66 ± 1.21	0.274	L1	DT	7.72 ± 1.34	9.08 ± 1.03	**0.033**
	IT	8.19 ± 0.91	8.76 ± 1.26	0.494		IT	8.42 ± 1.04	9.98 ± 2.68	0.274
	RT	15.75 ± 1.29	17.3 ± 1.49	0.091		RT	16.02 ± 1.29	18.94 ± 2.89	**0.026**
R2	DT	7.56 ± 1.63	8.88 ± 1.23	0.076	L2	DT	7.68 ± 1.55	8.89 ± 1.25	0.109
	IT	8.27 ± 1.24	9.09 ± 2.61	0.602		IT	8.13 ± 1.32	8.66 ± 1.16	0.602
	RT	15.71 ± 1.01	17.85 ± 2.67	0.076		RT	15.69 ± 1.14	17.43 ± 1.79	0.062
R3	DT	7.95 ± 1.16	9.54 ± 1.46	**0.041**	L3	DT	8.01 ± 1.38	9.55 ± 1.45	0.076
	IT	8.03 ± 0.61	8.45 ± 2.75	0.779		IT	8.07 ± 0.78	9.01 ± 2.49	0.602
	RT	15.85 ± 0.94	17.87 ± 2.53	**0.033**		RT	15.95 ± 1.05	18.43 ± 2.35	**0.015**
R4	DT	7.7 ± 1.39	9.74 ± 1.47	**0.02**	L4	DT	7.91 ± 1.5	8.88 ± 2.37	0.153
	IT	8.23 ± 0.79	8.51 ± 3.1	1		IT	7.99 ± 1.14	9.29 ± 3.13	0.841
	RT	15.81 ± 1.04	18.12 ± 2.6	**0.026**		RT	15.77 ± 1.02	18.05 ± 2.41	**0.009**

Values are presented as the mean ± SD unless otherwise specified. P values were calculated using Mann-Whitney U tests. Text in bold indicates statistical significance. The unit of the DT, IT and RT are [second].

R = right; L = left; SD = standard deviation; DT = decrease time, IT = increase time; RT = recovery time.

## Discussion

In this study, we utilized a wireless multi-channel CW-NIRS system and a beat-to-beat finger photoplethysmography device to investigate hemodynamic changes that occur during repeated squat-stand maneuvers. We compared SBP, DBP, MAP, and HR in a control group and an OI symptom group. We also evaluated the individual CA performance through statistical analysis of DHbT variation.

There were no between-group differences in the absolute levels and the variations of SBP, DBP, and MAP after the squat-stand maneuver. This may be because subjects had no history of OH, even though the symptom group had OI symptoms. However, significant variations in HR were observed in the symptom group after the squatting rest, which could indicate a compensatory effort by the heart to maintain cardiac output (CO) constant; this may further indicate impairment of the baroreflex function involved in the pathogenesis of IOH^21,22^. This suggests that the symptom group had a mildly impaired CA compared to the control group. In the same context, from the IOH diagnostic test, a few subjects demonstrated IOH-positive regardless of the presence of OI symptom. However, since the conventional diagnosis method is established for the supine-stand maneuver, the transient BP drop during squat-stand maneuver should employ a different diagnosis method for the categorization of the control and symptom group. It addresses the fact that the measured BP drop was moderate as compared to the previous investigation conducted to young healthy adults (SBP = 56 ± 15.7 mmHg and DBP = 37.0 ± 6.8 mmHg)^23^, which resulted in false-positive outcomes. The cardiovascular responses did not demonstrate clear evidence of degradation in the CA performance, since finger photoplethysmography was an indirect method to assess CA, which is an overlooking method that measures from the finger artery (i.e. a branch of the brachial artery).

**Figure 5:**
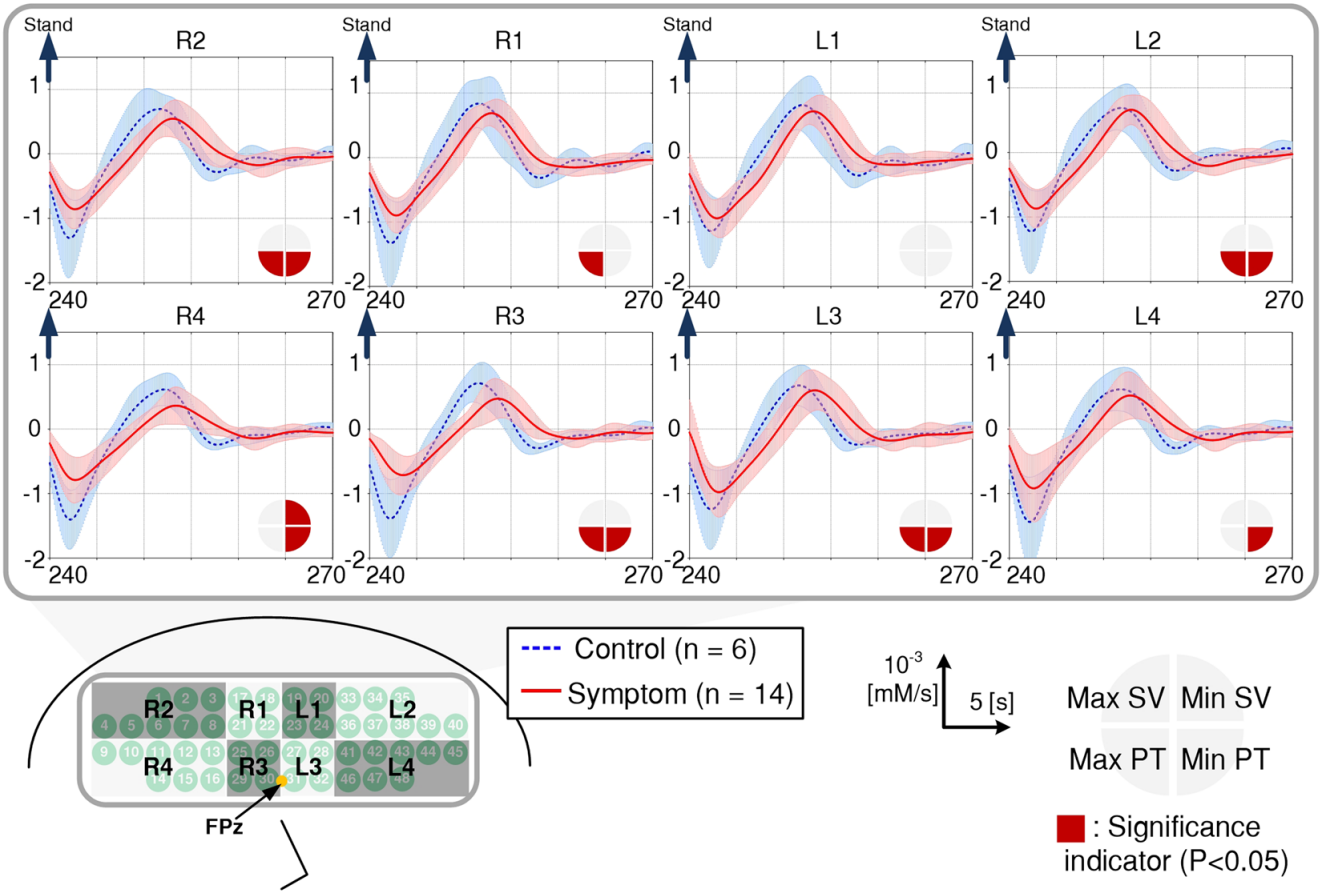
Transient grand-average of DHbT response between control and symptom group. The figure shows transient signal of grand-averaged DHbT of each groups. The blue and red lines indicate the grand-averaged waveform of the control and symptom groups. The shaded red and blue bands represent ± standard deviation. The quarter circle in the waveform represents significance, whereby filled red indicates statistical significance of each parameter (Max/Min SV, Max/Min PT) measured from DHbT. Fpz = frontal pole zero; SV = slope variation; PT = peak time.

Unlike BP and HR, the cerebral hemodynamic response, HbT, is directly related to CA^8,14^. HbT increases when a subject squats; the squatting-rest position results in increase in the venous return due to muscle contraction of the lower body^24^. When a subject stands, gravity pulls the venous blood towards the feet initiates the HbT drop, which might be associated with a decrease in CBF^25^. Accordingly, the baroreceptor activates to stimulate the vasomotor center to recover CBF as a compensatory mechanism that increases systemic vascular resistance or CO through autonomic-reflex activity^26^. It is well known that the relative change of CBV has a linear relationship with CBF^27,28^. Furthermore, the variations in the CBV and HbT are proportionally related^21^. As such, we revealed significant between-group differences in the DHbT response, which is the absolute change of HbT with respect to time, using a CW-NIRS device during the squat-stand maneuver. Our results clearly demonstrated increased PT in the entire sections except L1 and decreased SV in section R4 in the symptom group compared to the control group. The group averages of the DHbT response are shown in Fig. 5. The DHbT response showed a clear difference between the two groups, which shows that DHbT can be used as an objective parameter for the assessment of an individual’s vasoreactivity.

Meanwhile, the PT of the DHbT has demonstrated significant increase in the right hemisphere (especially in section R3) among the symptom group as compared from the control group. This result could reflect the hemisphere asymmetry in parasympathetic control of the heart^29^. Namely, previous studies have shown that sympathetic cardiovascular control is lateralized to the right hemisphere^30,31^. In addition, the lack of a right-sided HbT increase in response to pressure is indicative of an impaired vasoreactivity caused by dysfunction of the right hemisphere and the sympathetic nervous system^32^. Thus, the degraded fluctuation of the DHbT response in the right hemisphere is opening up the possibility of the lateralization of the sympathetic cardiovascular control in the brain.

Interestingly, both DT and RT extracted from HbT showed significant increase in the symptom group compared to the control group. This result clearly shows that the reach time to nadir was increased, which evidently shows the dysfunction of vasoreactivity in the symptom group right after squatting rest. However, since the results from DHbT demonstrates higher level of statistical significance than those from HbT, the CA assessment can be made more accurately with the metrics using DHbT than conventional schemes using HbT.

We tested the feasibility of using a multichannel CW-NIRS device as a CA performance assessment tool. The derivative of HbT, DHbT during the squat-stand maneuvers, demonstrated significant between-group difference, such as SV, PT of the DHbT response.

The utilization of CW-NIRS in CA assessment is preferred over a finger photoplethysmography-based BP monitoring scheme, due to its portability and high temporal and spatial resolution. As such, CW-NIRS devices can be used for the CA assessment in patients with neurological and psychiatric disorders. In addition, the proposed method can be used on healthy people on a regular basis to assess CA performance.

For future work, we plan to perform experiments with patients diagnosed with IOH or OH to verify the method by assessing the dysfunction of autonomous nervous system, where the present work was applied limitedly to young adults with OI symptoms. The comparison of the DHbT response between the healthy controls and clinically diagnosed patients will allow us to validate the proposed evaluation method with respect to its sensitivity and specificity.

## Data Availability

The datasets generated and/or analyzed during the current study are available from the corresponding author on reasonable request.
